# Supplementary material for: Proline/arginine dipeptide repeat polymers derail protein folding in amyotrophic lateral sclerosis
Source: Nat Commun. 2021 Jun 7;12:3396. doi: 10.1038/s41467-021-23691-y (PMC8184751; doi:10.1038/s41467-021-23691-y)
Supplement: Supplementary file 1 — Supplementary Information [file 41467_2021_23691_MOESM1_ESM.pdf]

Supplementary Information for

Proline/arginine dipeptide repeat polymers derail protein folding in amyotrophic lateral sclerosis

Maria Babu, Filippo Favretto, Alain Ibáñez de Opakua, Marija Rankovic, Stefan Becker and Markus Zweckstetter

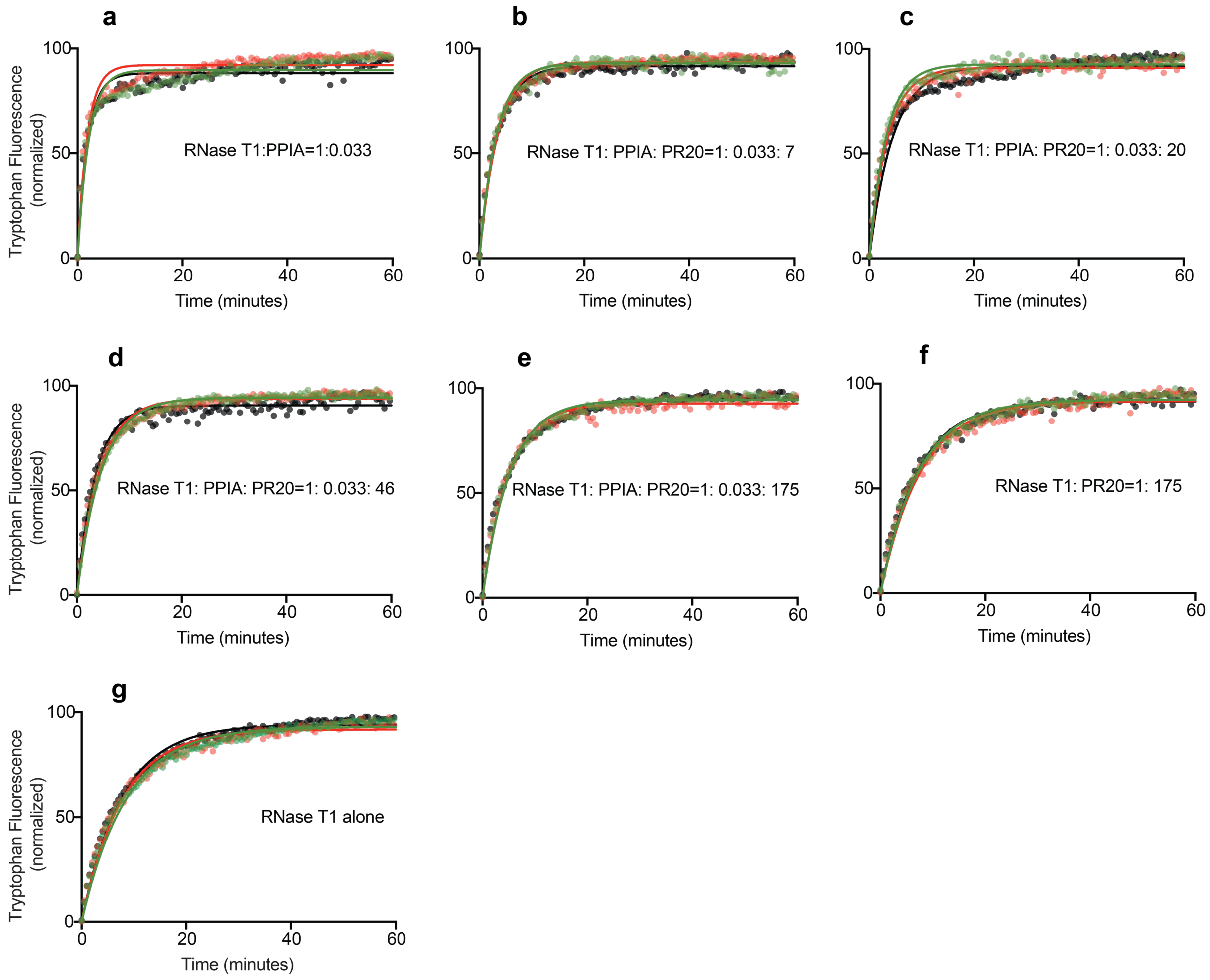

Supplementary Fig. 1 | Individual refolding curves of RNaseT1 corresponding to the average values shown in Fig. 1b. Green, pink and black dotted lines represent the three replicates of RNaseT1 folding under the conditions a) RNaseT1:PPIA=1:0.033, b) RNaseT1:PPIA:PR20=1:0.033:7, c) RNaseT1:PPIA:PR20=1:0.033:20, d) RNaseT1:PPIA:PR20=1:0.033:46, e) RNaseT1:PPIA:PR20=1:0.033:175, f) RNaseT1: PR20=1:175 and g) RNaseT1 alone. Mono-exponential fits corresponding to each replicate are shown in same colours using continuous lines.

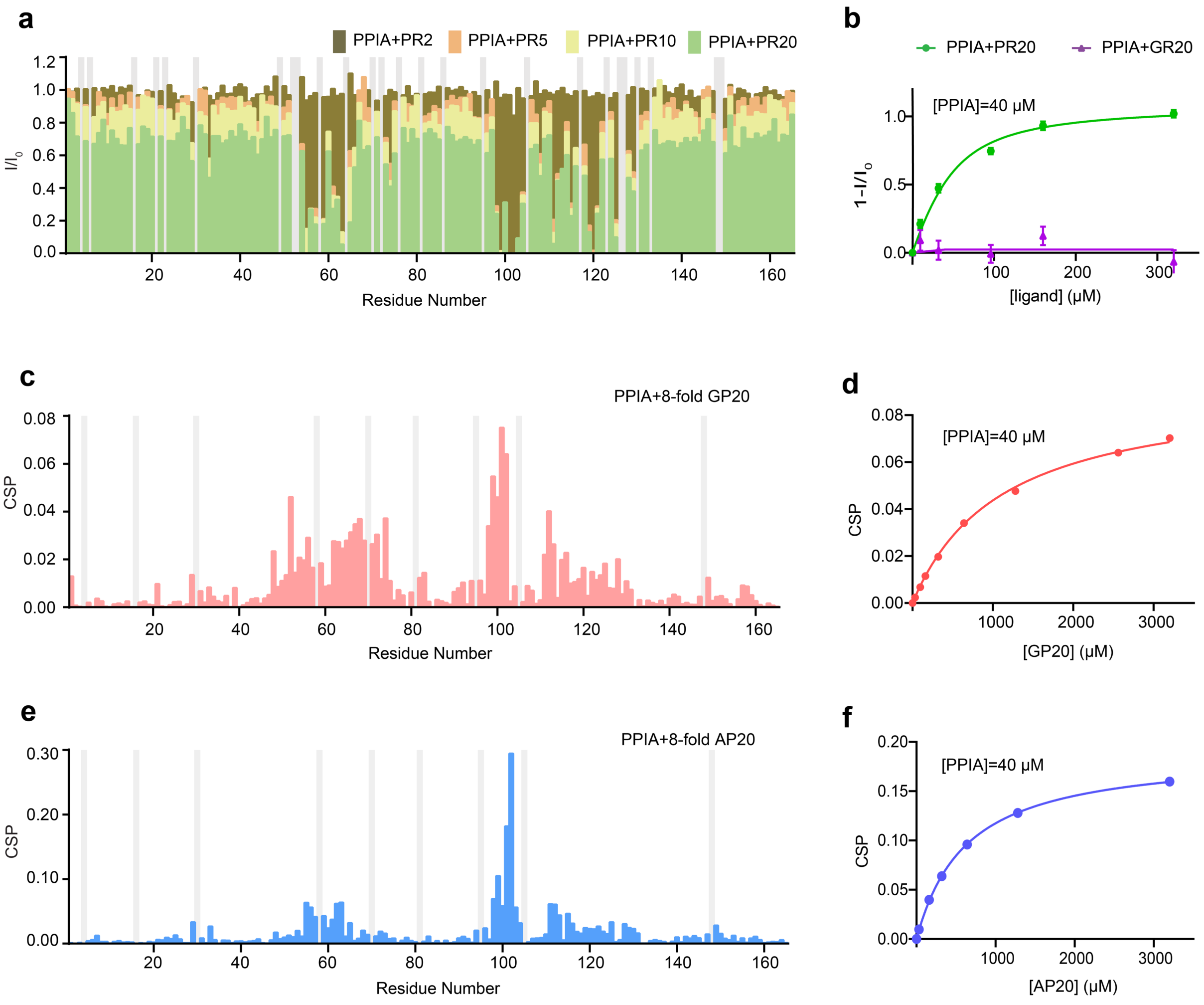

Supplementary Fig. 2 | Interaction of C9orf72-associated repeat polymers with PPIA. a, Residue-specific changes in the intensities of ^{1}H-^{15}N HSQC peaks of PPIA upon addition of 8-fold excess of PR20 (green), PR10 (yellow), PR5 (orange) and PR2 (brown). I and I_0 are the intensities of the PPIA HSQC cross peaks in the presence and absence of the PR repeat polymers, respectively. **b,** Intensity changes of the cross peak of Arg55 in PPIA as a function of increasing concentration of PR20 and GR20. The lines represent least-square fitting of experimental data from which K_d values were derived. Error bars represent the error in 1-(I/I_0) calculated according to equation 2. **c,** Averaged $^{1}H/^{15}N$ chemical shift perturbations (CSPs) of HSQC peaks of PPIA upon addition of 8-fold excess GP20. **d,** Averaged $^{1}H/^{15}N$ CSPs of Arg55 of PPIA for increasing GP20 concentrations. **e,** Averaged $^{1}H/^{15}N$ chemical shift perturbations (CSPs) of HSQC peaks of PPIA upon addition of 8-fold excess of AP20. **f,** Averaged $^{1}H/^{15}N$ CSPs of Arg55 of PPIA as a function of increasing AP20 concentration.

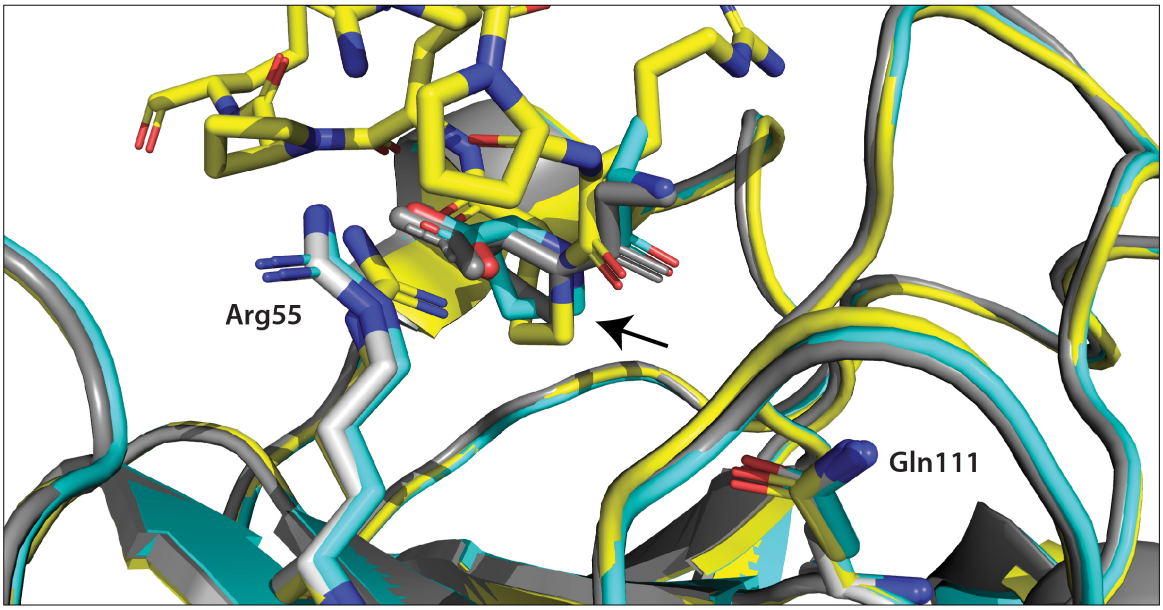

Supplementary Fig. 3 | Comparison of the structure of the active site of PPIA in complex with PR20 (this work; yellow) and Xaa-proline dipeptides (grey; PDB code 5CYH; https://www.wwpdb.org/pdb?id=pdb_00005cyh; cyan PDB code 2CYH; https://www.wwpdb.org/pdb?id=pdb_00002cyh)[1] . The side chain of Arg55, which is critical for substrate binding and catalytic activity of PPIA, is labelled. Gln111 of PPIA does not make direct contacts with the substrates, but is part of a dynamic network of residues in the binding pocket[2]. The proline of the substrate is marked by an arrow.

Supplementary Table 1. X-ray data collection statistics.

**Data statistics**	**PR20/CypA-complex**
Wavelength	1.0 Å
Beamline	SLS-X10SA
Detector	Dectris EIGER2 16M
Space group	$P6_1$
a	83.32 Å
b	83.32 Å
c	52.45 Å
α, β	90°
γ	120°
Resolution[a]	42.47-1.31 Å (1.34-1.31 Å)
Reflections measured	1,005,075
Unique reflections	49,424
Redundancy	20.06 (17.27)
Completeness(%)	98.7 (95.27)
Mean $I/\sigma(I)$	21.55 (1.6)
R_{int} (%)	6.56 (79.39)
R_{rim} (%)	3.7 (50)

[a]Values in parentheses are outer-resolution shell.

Supplementary Table 2. X-ray structure refinement statistics of the PR20/PPIA-complex.

R-factor	13.4%
R_{free}[a]	16.6%
Solvent	55.68%
Mean B-value (Å^2)	
chain A	28.32
chain B	30.44
waters	40.76
No. of protein residues	171
No. of water residues	163
**Root mean square deviations from ideal geometry**	
Bond lengths	0.019 Å
Bond angles	2.14°
**Ramachandran plot (%)**	
Favoured	93.75
Allowed	6.25
Outliers	0

[a]R_{free} was determined using 5% of the data.

Supplementary References

1. Schaller, T. et al. HIV-1 capsid-cyclophilin interactions determine nuclear import pathway, integration targeting and replication efficiency. *PLoS Pathog* **7**, e1002439 (2011).
2. Márquez, C.L. et al. Kinetics of HIV-1 capsid uncoating revealed by single-molecule analysis. *Elife* **7**, e34772 (2018).
